# An unusual case of giant cell myocarditis missed in a Heartmate-2 left ventricle apical-wedge section: a case report and review of the literature

**DOI:** 10.1186/1749-8090-8-12

**Published:** 2013-01-17

**Authors:** Kim Anderson, Michel Carrier, Philippe Romeo, Guy B Pelletier, Mark Liszkowski, Normand Racine, Michel White, Anique Ducharme

**Affiliations:** 1 Department of Medicine, Montreal Heart Institute, University of Montreal, 5000, Belanger East, Montreal, QC, H1T 1C8, Canada; 2 Department of Cardiac Surgery, Montreal Heart Institute, University of Montreal, 5000, Belanger East, Montreal, QC, H1T 1C8, Canada; 3 Department of Pathology, Montreal Heart Institute, University of Montreal, 5000, Belanger East, Montreal, QC, H1T 1C8, Canada

**Keywords:** Giant cell myocarditis, Lymphocytic myocarditis, Lymphoma, Immunosuppression, Heart transplant, Endomyocardial biopsy, Left ventricular assist device

## Abstract

Herein we present a case of fulminant myocarditis in a woman previously treated for B-cell lymphoma. While the clinical context was suggestive of adriamycin-induced cardiomyopathy, the initial pathology of the Heartmate-2 apical core showed lymphocytic myocarditis. After 8 months of stability, the patient presented with progressive heart failure and recurrent ventricular arrhythmias. An endomyocardial biopsy revealed findings typical of giant cell myocarditis (GCM); poor response to immunosuppressive therapy and marked hemodynamic instability led to urgent transplantation. To our knowledge, this is the first reported case of GCM following an acute lymphocytic myocarditis and the second GCM case associated with B-cell lymphoma.

## Background

Giant cell myocarditis (GCM) is a rare form of fulminant myocarditis which has a poor outcome without heart transplantation [1]. The involvement of T-lymphocytes may explain the common association with other autoimmune conditions and the potential role of immunosuppressive therapy in GCM [1,2]. We describe the challenging case of a woman previously treated for B-cell lymphoma who presented to the emergency department in cardiogenic shock. While the clinical context suggested adriamycin-induced cardiomyopathy, the Heartmate-2 apical core pathology showed lymphocytic myocarditis. After initial improvement on mechanical support, she presented with recurrent heart failure symptoms 8 months later; a RV endomyocardial biopsy was performed and revealed GCM. Immunosuppressive therapy was initiated but persistent RV failure and arrhythmic storm led to urgent transplantation.

This clinical scenario illustrates the need for a better understanding of the pathophysiology of fulminant myocarditis, in order to improve diagnostic and treatment of this life-threatening condition. In particular, this case raises many important issues, including 1) the potential relationship between GCM and lymphocytic myocarditis; 2) the possibility that the large LV apical specimen could have missed GCM; and 3) the presence of atherosclerosis lesions in the donor heart.

## Case presentation

**Figure 1:**
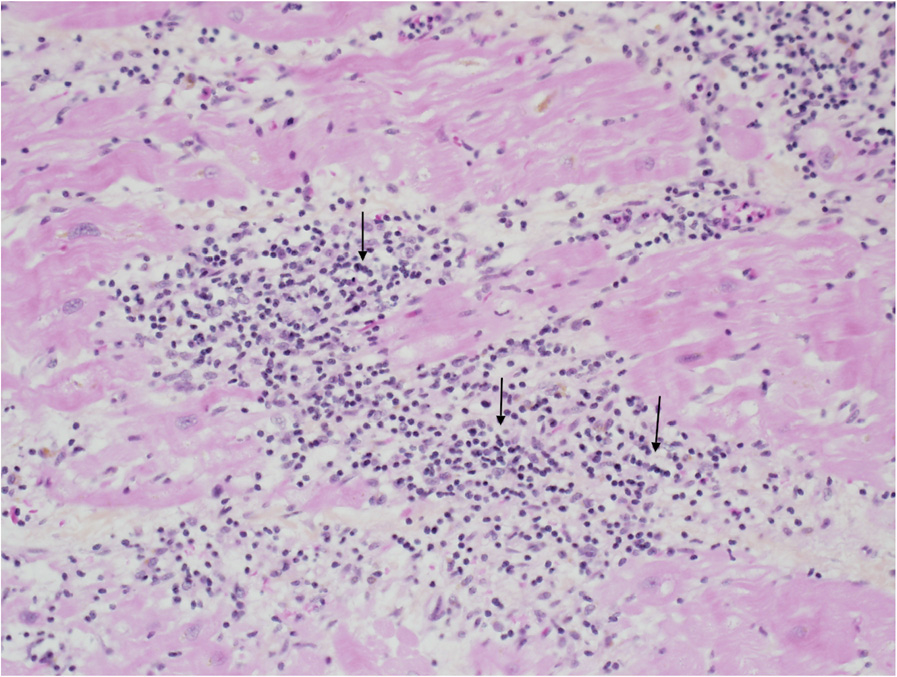
**Left ventricular apical core specimen. **Interstitial inflammatory infiltrate rich in lymphocytes (arrows) in an area of myocyte loss associated with peripheral myocyte injury (HPS, 20X).

A 41 years old woman was transferred to our hospital in cardiogenic shock. Fourteen months earlier she has been treated for a cutaneous centro-follicular type-B lymphoma with R-CHOP (total adriamycin dose of 300 mg/m^2^) and has well recovered since. Echocardiography at presentation showed a dilated left ventricle (LV), left ventricular ejection fraction (LVEF) of 10%, and preserved right ventricle (RV) function; the coronary angiography was normal. She was initially supported by an Impella-2.5L, followed by a Heartmate-II five days later. Histology of the LV-apical core section showed multifocal interstitial inflammatory infiltrates, predominantly lymphocytic, with edema and extensive mycocytes destruction, without giant cell or extensive necrosis (Figure 1). Immunohistochemistry revealed a T-cell phenotype, different from the previous lymphoma and immunofluorescence was suggestive of acute lymphocytic myocarditis. She received a defibrillator and was discharged on standard heart failure therapy. She improved on Heartmate-II support with minimal symptoms.

**Figure 2:**
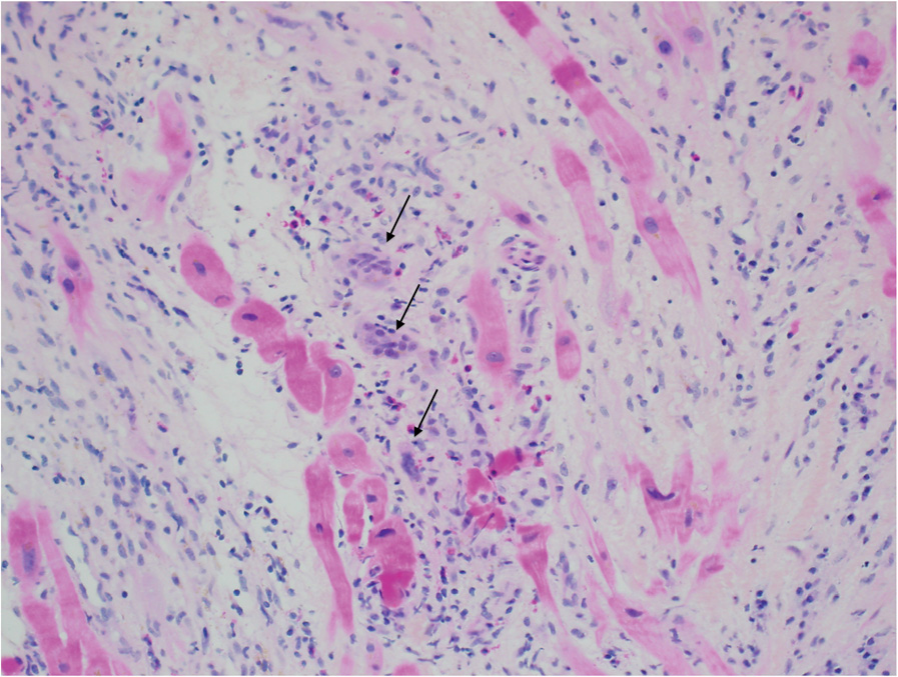
**Right ventriclular endomyocardial biopsy. **Mixed inflammatory infiltrate with giant cells (arrows) in a background of severe myocytes dropout and features of granulation tissue (HPS, 20X).

Eight months later she was readmitted after two weeks of congestive symptoms; NT-proBNP (3421 ng/L, from 1454 in June) and troponins T (0,58 mcg/L, normal ≤ 0.03 mcg/L) were increased and echocardiography showed a decline in LVEF (30% from 45% 2-months prior), absence of aortic valve opening and a dilated and hypokinetic RV. An endomyocardial biopsy (EBM) revealed severe mycocytes’ dropout, associated primarily with granulation tissue, a mixed inflammatory infiltrate and the presence of multinucleated giant cell with a non-myogenic appearance (Figure 2), all suggestive of GCM. Immunosuppressive therapy was initiated with high dose corticosteroids and cyclosporine [1]. Unfortunately, she developed incessant ventricular tachycardia and profound RV failure requiring amiodarone and milrinone; she underwent urgent transplantation 16-days later.

**Figure 3:**
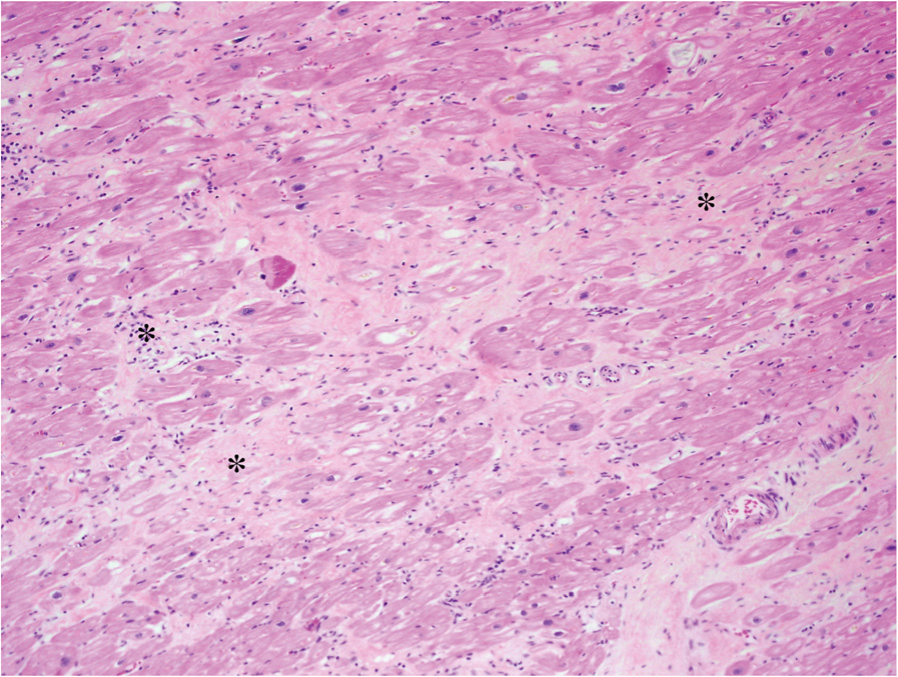
**Native heart explants: Left ventricular section. **Extensive interstitial fibrosis with residual areas of granulation tissue and mild lymphocytic infiltrates (HPS, 10X).

**Figure 4:**
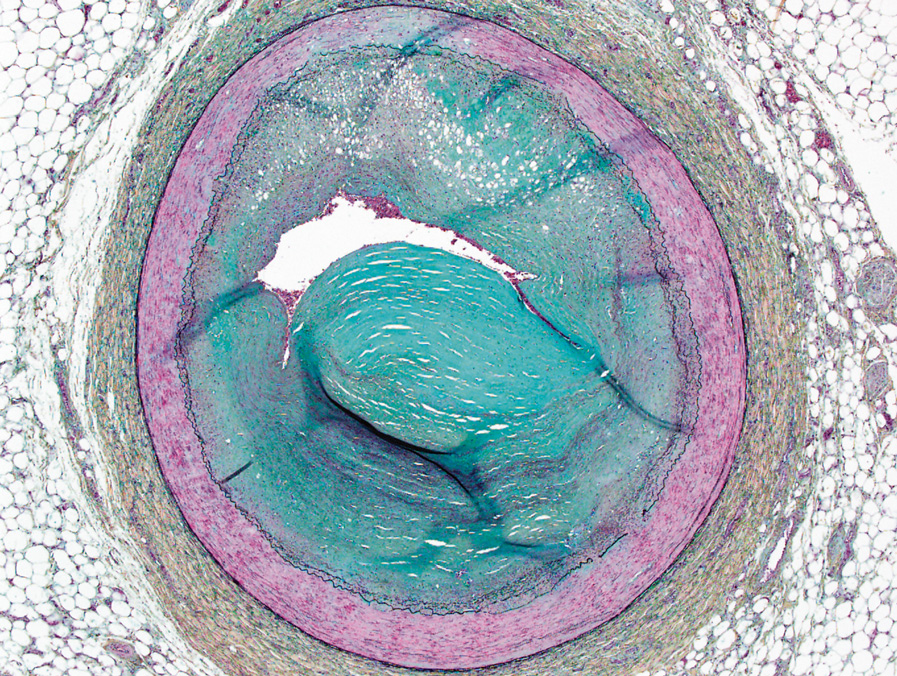
**Autopsy explants of the donor heart, section of the mid left anterior descending coronary artery. **Severe concentric intimal atherosclerotic plaque with significant luminal stenosis (Movat, 20X).

Histopathology of the explanted heart showed diffuse and irregular interstitial fibrosis, without extensive necrosis, associated with residual areas of granulation tissue, mild to moderate lymphocytic infiltrates and mild foci of myocytes damage (Figure 3). The post-operative course was complicated by persistent shock, requiring right side extracorporeal life support, an intra-aortic balloon pump and continuous renal replacement therapy. Immunosuppressive regimen comprised of rabbit antithymocyte globulin (RATG) induction, corticosteroids, mofetilmycophenolate, basiliximab and later tacrolimus. Unfortunately, she died of multi-organ failure one month after transplantation. At autopsy, no signs of giant cell or hyperacute rejection could be found; however, there was evidence of sub-acute myocardial ischemia and severe three-vessel disease (Figure 4), in spite of a reported ‘normal’ coronary angiogram at the donor site.

## Discussion

The clinical presentation of GCM is usually dramatic, but some may present an indolent course with presence of symptoms for months to years before the proper diagnosis is made [3]. Ventricular arrhythmias are initially found in 14% of patients, but will develop in 50% of the patients; fascicular and high degree atrioventricular blocks are also frequently encountered [1]. The median survival without heart transplantation is poor, between 3.0 to 5.5 months from the onset of symptoms [1,3], and relapse in the transplanted graft may occur in up to 25% of the cases [1-3].

Histopathology is characterized by the presence of multinucleated giant cells, with diffuse myocardial necrosis in the absence of granulomatous myocarditis; fibrosis may be prominent. The precise pathophysiology is unknown, but seems to involve a T-lymphocytes-mediated autoimmune process, which may explain the common association (up to 20%) with other autoimmune disorders, typically inflammatory bowel diseases and tumors, mostly thymoma [1,2]. Association with lymphoma has been described twice in the literature. One patient presented in cardiogenic shock; multi-organ infiltration (including the myocardium), with centrocytic lymphoma cells, myocytolysis and extensive fibrosis associated with a mixed cellular infiltrate including giant cells were seen at autopsy [4]. Another case occurred in the context of autologous bone marrow transplantation with IL-2 activated stem cells. Autopsy revealed myocardial infiltration with T-helper lymphocytes and apoptosis of myocytes. The authors suggested that IL-2 might have stimulated a preferential activation of T-helper lymphocytes resulting in an autoimmune process manifested as GCM [5].

Kodama and colleagues developed an experimental model of GCM using Lewis rats inoculated with human cardiac myosin fragments and sacrificed at 14 and 84 days. They showed typical myocarditis with multinucleated giant cells and diffuse fibrosis in the early phase, with disappearance of the mononuclear infiltrates in the later phase suggestive of healed myocarditis. Anti-cardiac myosin antibodies were significantly higher in the groups sacrificed earlier, but all had higher titers than controls [6]. These findings could be prevented by cyclosporine [7], FK-506 (Tacrolimus) [8] and anti-alphabeta [9] T-cell receptor antibodies, supporting the role of a T-cell mediated autoimmune process in GCM.

Clinically, immunosuppressive therapy might improve survival in GCM, in contrast with other types of myocarditis [10]. In a retrospective study, GCM patients who received immunosuppression (corticosteroids and cyclosporine, with/without azathioprine) had an improved survival without cardiac transplantation (12.3 versus 3.0 months (p=0.001)), while corticosteroids alone had no effect [1]. An observational study of biopsy-proven GCM patients (excluding those with fulminant presentation) and symptoms of heart failure or arrhythmia reported that treatment with cyclosporine and corticosteroids for one year, with/without muromonad-CD3 pre-treatment for ten days dramatically improved survival (64%) without cardiac transplantation [2].

Whether there was a relationship with lymphocytic myocarditis or if its presence could have been a contributing factor to GCM development in our patient remains unclear. The reported sensitivity of RV-EMB in adults is relatively low for a precise diagnosis of myocarditis, from 10-67% [10], but seems better for GCM, up to 80% [11], despite significant interobserver variability [1]. Predictors of the absence of giant cell in the explanted heart after a positive EMB include long delay between the EMB performance and the explanted heart’s pathologic analysis and symptoms duration [11]. The possibility that the large LV core specimen could have missed GCM is unlikely but whether the lymphocytic infiltrates may have triggered an inflammatory response leading to development of giant cells infiltrates remains unanswered. Another diagnosis possibility included the recurrence of lymphoma, but immunohistochemistry depicted a different phenotype of T-cell infiltrate; also, while we cannot provide evidence that the granulomas seen on the RV biopsy specimen were not secondary to an immune response to infection or inotropic drugs, this is highly improbable, given the fact that she was not on inotropes at the time of RV-EBM and that not infectious agents were found despite extensive workup. Lastly, differentiating GCM from a granulomatous myocarditis can be difficult [12], but the fulminant presentation, the absence of atrioventricular block and the poor outcome are more typical of GCM [13].

The last lesson learned from this case were the autopsy findings of severe coronary atherosclerosis in the transplanted heart. As transplant cardiologist, we are often facing critical decision making such as to reject/accept a donor heart for our critically ill patients. Unfortunately, we sometimes rely on incomplete or erroneous information, as was the so-called “normal angiogram” report from the referring center. Having known the real extent of CAD (revealed at autopsy) in this donor heart, we would have gone without transplantion, significant donor coronary atherosclerosis being a major risk for early graft failure, with reported 30-day mortality of 7.5% with single-vessel involvement and 42.3% with multiple-vessel disease [14].

## Conclusions

This case report illustrates important learning points. First, the bimodal presentation of initial improvement followed by decompensation is unusual for GCM. Also, the presence of GCM may not always be appreciated at the initial biopsy, even though a large sample is provided, such as a Heartmate-II apical core; therefore, it depicted the importance of obtaining a precise histological diagnosis since immunosuppressive therapy might improve survival. One question remains unanswered: Was the initial diagnosis really an acute lymphocytic myocarditis or was it the indolent phase of a GCM? Could lymphocytic myocarditis have been a contributing factor to development of GCM? Further research is needed to better understand the pathophysiology and treatment in this rare but life-threatening disease.

## Consent

Written informed consent was obtained from the patient's husband for the publication of this Case report and the accompanying pictures. A copy of the written consent is available for review at the Editorial office of this journal.

## Abbreviations

GCM: Giant cell myocarditis; LV: Left ventricle; LVEF: Left ventricular ejection fraction; RV: Right ventricle; EBM: Endomyocardial biopsy.

## Competing interests

All authors declare that they have no competing interest.

## Authors’ contributions

KA participated directly in the care of the patient and drafted the manuscript. MC participated directly in the care of the patient and participated in the writing of the manuscript. PR analyzed pathologic specimen and participated in the writing of the manuscript. GBP participated directly in the care of the patient and participated in the writing of the manuscript. ML participated directly in the care of the patient and participated in the writing of the manuscript. MW participated directly in the care of the patient and participated in the writing of the manuscript. NR participated directly in the care of the patient and participated in the writing of the manuscript. AD participated directly in the care of the patient, supervised KA both clinically and in the writing of this manuscript. All authors read and approved the final manuscript.
